# Influence of Anxiety/Depression, Age, Gender and ASA on 1-Year Follow-Up Outcomes Following Total Hip and Knee Arthroplasty in 5447 Patients

**DOI:** 10.3390/jcm10143095

**Published:** 2021-07-13

**Authors:** Julia Sabrina Götz, Achim Benditz, Jan Reinhard, Melanie Schindler, Florian Zeman, Joachim Grifka, Felix Greimel, Franziska Leiss

**Affiliations:** 1 Department of Orthopedics, University Medical Center Regensburg, Asklepios Klinikum Bad Abbach, Kaiser-Karl-V.-Allee 3, 93077 Bad Abbach, Germany; ju.goetz@asklepios.com (J.S.G.); Achim.Benditz@ukr.de (A.B.); j.reinhard@asklepios.com (J.R.); m.schindler@asklepios.com (M.S.); j.grifka@asklepios.com (J.G.); f.leiss@asklepios.com (F.L.); 2 Center for Clinical Studies, University Medical Center Regensburg, Franz-Josef-Strauss-Allee 11, 93053 Regensburg, Germany; florian.zeman@ukr.de

**Keywords:** anxiety, depression, knee arthroplasty (TKA), hip arthroplasty (THA), total joint arthroplasty (TJA), postoperative outcomes

## Abstract

Introduction: There are many factors influencing the outcome after total joint arthroplasty (TJA). In particular, patient-related factors such as age, gender, ASA (American Society of Anesthesiologists), or preoperative anxiety/depression have become increasingly important. The aim of this study was to examine the association of these parameters with 1-year postoperative outcomes after total knee and total hip arthroplasty (TKA, THA). Methods: A retrospective cohort of 5447 TJA patients was evaluated by pre- and postoperative analysis of EQ-5D, EQ-VAS and WOMAC Score. Furthermore, major focus was put on the association between age, gender, ASA, preoperative anxiety/depression and outcome parameters. Results: 53.3% (2903/5447) of all patients were identified with anxiety/depression at time of surgery. In the analysis, patients without anxiety/depression showed statistically significantly (*p* < 0.05) better EQ-5D, EQ-VAS and WOMAC scores. In addition, patients with ASA 2 or 3 and age over 70 years showed statistically significantly (*p* < 0.01) worse EQ-5D and WOMAC scores. Gender did not influence the postoperative EQ-5D and WOMAC results, but men had significantly better EQ-VAS scores than women in this study. Conclusion: Preoperative anxiety/depression symptoms show worse clinical outcomes 1 year postoperatively after TJA. Other outcome-influencing factors are higher age and ASA 2 or 3. In the future, such patients should be identified, and as far as applicable, a treatment of anxiety/depression or comorbidities should be implemented preoperatively of the surgical procedure to improve clinical outcomes.

## 1. Introduction

An estimated 250 million people globally suffer from osteoarthritis, a common chronic musculoskeletal condition [1]. The incidence of osteoarthritis is rising due to overaging, an increasingly overweight society and joint injuries [2]. Symptoms in the affected joint/s such as stiffness, pain and instability, tend to worsen in severity over time [3]. These symptoms can lead to functional limitations, especially considering mobility, as well as decrease of independence in activities of daily living [3,4,5]. After a certain point, conservative interventions are unsuccessful in restricting disease progression of osteoarthritis [5]. Therefore, in order to improve signs and symptoms of osteoarthritis, the most common orthopedic procedures are elective primary total hip and knee arthroplasty (THA, TKA). 

Depressive symptoms such as decreased cognitive functioning, including difficulties in concentrating and making decisions, life-interfering disruptions to mood and additional reduced motivation to engage in behavioral activity, are common among patients with osteoarthritis [6,7]. In addition to depression, anxiety also plays a major role. The broad term “anxiety” is used to describe a range of anxiety-related symptoms. Among others, it includes cognitive, emotional or physiological symptoms [8]. Additionally, psychological comorbidities are associated with increased hospital length-of-stay, return-to-theater and readmission rates [2,9,10,11].Thus, social burden is not only caused by the rise in hospital costs but also by premature retirement and loss of employment [9]. Patients demonstrating preoperative anxiety and depressive symptoms appear at greater risk of adverse outcomes, including worse pain, greater stiffness and poorer function following TKA and THA [12,13]. 

Increased age, medical comorbidities, higher body mass index (BMI) or reduced preoperative mobility are known factors linked to poorer outcomes [14,15]. Before TJA, women in general have worse functional parameters, suggesting that women are operated at an advanced stage of osteoarthritis [16]. Significantly higher rates of intraoperative and postoperative complications after THA, such as transfusions, prolonged intensive care unit (ICU) stay, and total length of stay (LOS), are detected in patients preoperatively classified as ASA III and IV compared to ASA I and II classified patients [17]. Younger patients <55 have higher improvements in WOMAC and EQ-5D scores 3 months postoperatively after primary THA or TKA [18]. Schwartz et al. predict an increasing incidence of revision total knee arthroplasty (78–182%) and revision total hip arthroplasty (43–70%) by 2030 in the 55–64 and 65–74 age groups, but a decreasing incidence of revisions in the 75–84 age group [19]. 

Studies, systematic reviews and meta-analyses investigate the relationship between age, gender, ASA or preoperative anxiety/depression and TKA or THA outcomes, but so far there has never been a study on all factors on the same study population. Furthermore, most studies only included small case numbers. We aimed to determine whether there is an association between age, gender, ASA or anxiety/depression at the time of surgery and outcomes at one year post-surgery following primary elective total hip and total knee arthroplasty.

## 2. Materials and Methods

**Figure 1:**
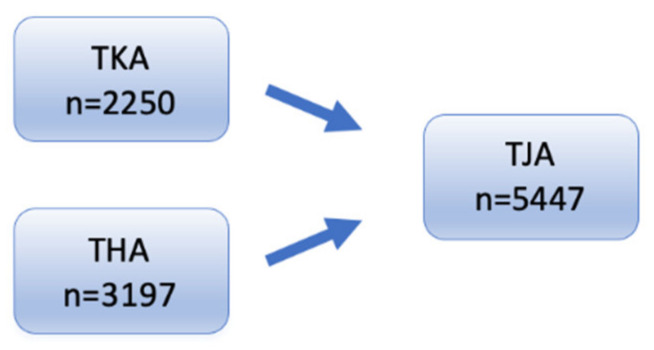
A total of 5447 included patients after TJA from 2013 to 2020. TKA = total knee arthroplasty/THA = total hip arthroplasty.

This retrospective cohort study evaluates outcomes after primary THA and TKA in patients with osteoarthritis. Using data from our hospital information system (ORBIS, Dedalus HealthCare, Bonn, Germany) we performed an analysis to investigate the influence of age, gender, ASA or preoperative anxiety/depression status on post-operative patient-reported outcomes (EQ-5D and WOMAC Score) after 1 year in 5447 consecutive patients between 2013 and 2020 after TJA, 3197 THA and 2250 TKA (Figure 1). All patients received WOMAC and Euroqol by mail one year postoperatively with the request to return it. Then, the data were transferred to the hospital information system. The study was approved by the local Ethics Committee with the approval number 21-2312-104 (IRB approval). The study was applied in accordance with the ethical standards of the Declaration of Helsinki 1975.

Inclusion criteria for the study were age over 18 years, primary cementless THA using an anterolateral approach and primary cruciate retaining cemented TKA. Exclusion criteria for the study were revision THA/TKA and indications for surgery other than osteoarthritis, such as avascular necrosis, fracture and malignancy. Another exclusion criterion was an incomplete questionnaire. All data were extracted from digitized patient records. General data such as age, gender and ASA-score (American Society of Anesthesiologists) were assessed. For adequate comparison to the literature, age was divided into three age groups: under 55, 55 to 70 and over 70 years. The common ASA physical status classification system was used to evaluate predicting perioperative risks. ASA is subdivided into 6 categories: a normal healthy patient (ASA I), a patient with mild systemic disease (ASA II), a patient with severe systemic disease (ASA III), a patient with severe systemic disease that is a constant threat to life (ASA IV), a moribund patient who is not expected to survive without the operation (ASA V) and a declared brain-dead patient whose organs are being removed for donor purposes (ASA VI). Correlation analysis of age, gender and ASA were carried out as part of the retrospective evaluations. The preoperative EQ-5D was used to identify patients with and without concomitant diagnosis of anxiety/depression who underwent TJA at our institution. In the standardized EQ-5D score, anxiety/depression was subdivided into 5 categories: not anxious/depressed, a little anxious/depressed, moderately anxious/depressed, very anxious/depressed and extremely anxious/depressed. The EQ-5D provides a description of five dimensions of health status ranging from 1 (full points in all categories), indicating full health, to 0 (no points in all categories), indicating dead. Furthermore, EQ VAS was collected. The patients had to rate on a scale of 0–100 points, 0 represents very poor and 100 very good health. The Western Ontario and McMaster Universities Osteoarthritis Index (WOMAC) is ordinal scaled on a scale from 0 to 4 (none, mild, moderate, severe, extreme). A worse result corresponds to a higher point value. The range of scores for the respective subscales are pain 0–20 points, stiffness 0–8 points and physical function 0–68 points. The standardized scores EQ-5D and WOMAC were recorded routinely pre- and one year (12 months) postoperatively. The operation time is defined between cut and suture of THA/TKA-procedure.

### Statistical Analysis

For descriptive analysis, mean values and standard deviation or median and interquartile range (q1, q3) are presented for continuous variables as well as absolute and relative frequencies for categorical variables. A Kruskal–Wallis Test was used to compare the EQ-5D and WOMAC score between preoperative factors (depression/anxiety, age groups, ASA score, gender). A *p*-value < 0.05 was considered statistically significant for all tests. All analyses were performed using SPSS 26.0 (IBM SPSS Statistics, IBM Corp., Armonk, NY, USA).

## 3. Results

We included 5447 patients after TJA from 2013 to 2020, 3197 THA and 2250 TKA (Figure 1). After excluding patients with incomplete questionaries postoperatively, 3594 with completed 1-year follow-up EQ-5D and 2543 WOMAC were evaluated. The mean age of the study patients was 66.58 years (SD 10.22; min 21; max 96) and the relative (absolute) frequency of gender was 43.3% (2359) men 56.59% (3088) women. Relative and absolute frequencies of the ASA-score are shown in Table 1. A total of 46.7% (2544/5447) of all patients were identified as being without anxiety/depression. In contrast, 9.5% (520/5447) of the patients were identified as very or extremely anxious/depressed at the time of surgery, while 25.8% were a little anxious/depressed (1403/5447) and 18.0% were moderately anxious/depressed (980/5447).

### 3.1. EQ-5D and EQ VAS

**Figure 2:**
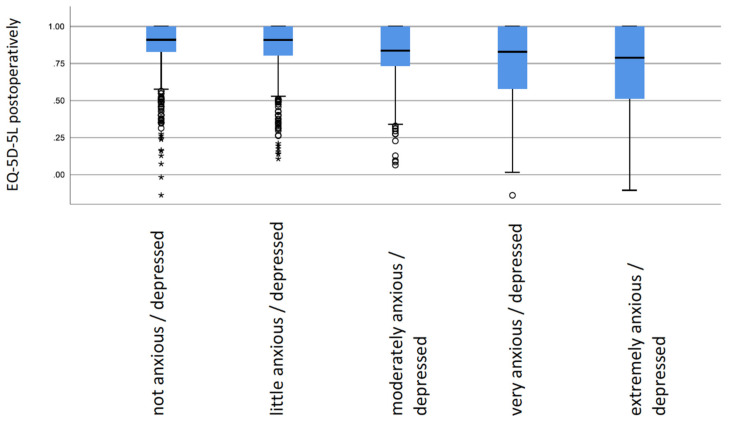
Boxplot: EQ-5D postoperatively according to preoperative anxiety/depression status.* are outliers.

The extent of anxiety/depression had a significant impact on EQ-5D after 1 year (*p* < 0.01). With increasing anxiety/depression, quality of life decreased from 0.91 to 0.79 (Table 2 and Figure 2). No significant gender differences regarding EQ-5D were found. In contrast, men had significantly better scores in the EQ-VAS than women.

There is a significant age correlation regarding the results of EQ-5D. Patients over 70 years showed a significantly (*p* < 0.01) worse result than patients between 55–70 years and patients under 55 years. The same applies to EQ-VAS. Patients over 70 years reported significantly poorer health than patients between 55–70 years (*p* < 0.01) and under 55 years (*p* < 0.01) of age (Table 3). Patients with a higher ASA-score showed worse clinical outcome scores. Patients with an ASA I had a significantly (*p* < 0.01) better EQ-5D outcome than patients with ASA II and III. Furthermore, patients with ASA II had a significantly better EQ-5D outcome than ASA III. In terms of EQ-VAS, and patients with ASA I had a significantly (*p* < 0.01) better result than patients with ASA II and III. Patients with ASA II had a significantly better result than patients with ASA III. In Table 3, an overview of mean values and standard deviations can be found. 

### 3.2. WOMAC Score

**Figure 3:**
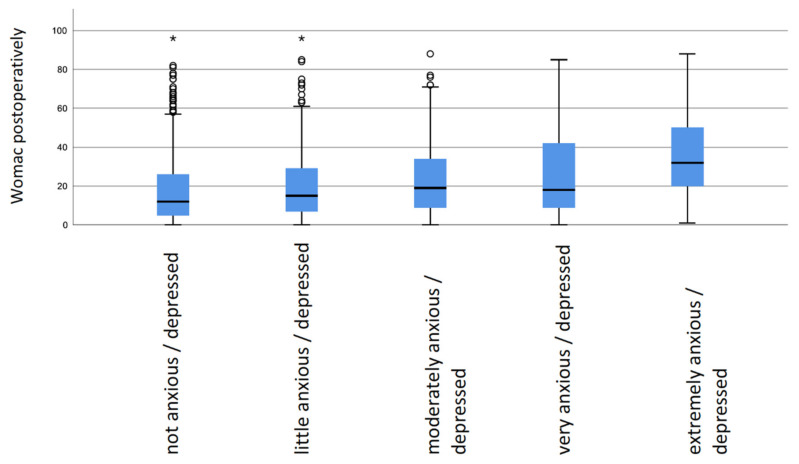
Boxplot: Western Ontario and McMaster Universities Osteoarthritis Index (WOMAC) postoperatively in dependence of preoperative anxiety/depression status. * are outliers.

Regarding WOMAC Scores, the results showed a similar pattern. Patients without anxiety/depressive symptoms preoperatively had the lowest WOMAC Score 12 months postoperatively, while scores significantly increased with increasing anxiety/depressive symptoms (*p* < 0.01) (Table 4 and Figure 3).

An overview of gender, age and ASA is shown in Table 3. No significant gender differences regarding the WOMAC Scores were found. In terms of WOMAC-Pain, those over 70 years of age had a significantly poorer outcome compared to those of 55–70 years (*p* < 0.01). Both patients under 55 years (*p* < 0.01) and patients 55–70 years (*p* < 0.01) showed a significantly better outcome in terms of WOMAC-Function and WOMAC-Total compared to the patients over 70 years old (Table 4), whereas no significant difference between the age groups 55–70 years and under 55 years were detected regarding WOMAC-Pain, WOMAC-Function and WOMAC-Total. Regardless of the age group under 55, 55–70 or over 70 years, there was no significant correlation in WOMAC-Stiffness.

A significant better outcome with ASA I compared to ASA II and ASA III applies to WOMAC-Pain (*p* < 0.01), WOMAC-Stiffness (*p* < 0.01), WOMAC-Function (*p* < 0.01) and WOMAC-Total (*p* < 0.01).

## 4. Discussion

We found a high prevalence of anxiety/depressive symptoms in a population with end-stage hip and knee osteoarthrosis in the present study. The present study determines the high impact of anxiety/depression, age and ASA on one-year postoperative outcomes after THA/TKA.

Total knee and hip arthroplasty (TKA, THA) are commonly used elective surgical treatments for end-stage osteoarthritis, demonstrating high success rates when assessed by objective medical outcomes. However, a considerable proportion of TKA and THA patients report significant dissatisfaction postoperatively, related to enduring pain, functional limitations and diminished quality of life, possibly affected by numerous factors including the mental health status of patients [20,21]. Anxiety and depression are broad terms used to describe a range of fear-related symptoms across a spectrum of severity. Our results show that 53.3% (2903/5447) of the patients had anxiety/depressed symptoms, while 9.5% (520/5447) of the patients were identified very or extremely anxious/depressed at the time of surgery. Contrary Rasouli M.R. et al. identified 12.7% and 6.4% of knee and the hip arthroplasty patients with concomitant depression or anxiety [22]. It should be noted that they did not specify how pronounced the patient’s anxiety/depression was. Regarding the global population, 7.2% have anxiety/depression disorders [23]. Furthermore, it has been stated that the population prevalence of depression and anxiety increases significantly each year [24]. Therefore, it can be concluded that the 9.5% “very or extremely anxiety/depressed” in the EQ-5D corresponds to the international average. The remaining 43.8% who indicate mild or moderate anxiety could be related to fear of surgical intervention and complications. No orthopedic study dividing by cause of anxiety or by when it originated was found. Depression and anxiety are not only predictors of worse clinical outcomes but also of increased complications [12,25].

In addition, Ali A. et al. emphasize that anxiety/depression is an important predictor for dissatisfaction compared to patients without anxiety/depression after TJA [6]. Our evaluations confirms that anxious/depressive patients have significantly worse WOMAC and EQ-5D score (*p* < 0.01) after TJA. Preoperative anxiety or depression leads to 6 times higher risk of being dissatisfied in contrast to patients with no anxiety or depression (*p* < 0.01) after total knee arthroplasty. Pain and function are important subjective outcome parameters. Several studies have demonstrated stronger pain, chronic pain and smaller improvements in pain as a predictive value in patients suffering from depression/anxiety preoperatively after primary TJA. In addition, these patients had significantly more functional limitations, instability and stiffness [12,26,27,28].

Besides poor clinical outcome, preoperative anxiety in THA increases the risk of postoperative delirium [29]. Anxiety and depression not only influence individual patient recovery but also hospital and personal costs and those incurred by premature retirement and loss of employment [22]. Not only in primary TJA but also during revisions, preoperative anxiety and depression significantly (*p* < 0.01) lead to an extended length of stay and higher 90 day readmission, 90 day emergency department visit or revision surgery, accompanied by increased costs [30]. Based on our study results, it is important to identify and treat patients with anxiety/depression preoperatively to achieve a better clinical outcome, minimize complications and reduce costs.

Chaturvedi R. et al. emphasize the high risks for ASA IV patients. The rate of postoperative complications, 30-day readmission, and 30-day mortality may be too high to justify surgery [31]. Furthermore, the importance of follow-up visits for all patients is emphasized, with special attention to patients with poor physical health conditions. However, patients classified as ASA III seem to be less adherent to follow-up visits than those with ASA II [32]. Mannion et al. explained that a higher ASA is not only associated with more complications but also with a worse patient-rated outcome 12 months after THA [33]. Our evaluation underlines that comorbidities measured with ASA Score are not automatically associated with worse postoperative outcomes in TJA. ASA II and ASA III patients had significantly worse EQ-5D, EQ-VAS and WOMAC scores than ASA I patients. However, there was no significant correlation with ASA IV patients. The American ASA comorbidity indices can be easily obtained for TJA patients and may assist with preoperative counseling regarding individual risks and benefits.

Women have a higher prevalence of osteoarthritis, worse symptoms and greater disability with similar radiographic severity compared to men [34,35,36,37]. In terms of gender differences after TJA, there is some evidence that women report more pain and reduced activities of daily living postoperatively compared to men [38,39]. On the one hand, Patek A.P. et al. verify female gender as an significant (*p* < 0.01) independent risk factor for wound infection, reoperation and readmission after THA. On the other hand, male gender was reported to be an independent risk factor for complications after TKA [40]. Significant (*p* < 0.01) independent risk factors for readmission and reoperation are female gender after THA and male sex after TKA [40]. Overall, the exact correlation is unknown. Our results refer only to the subjective evaluation of the patients. There are no significant gender differences in the EQ-5D or WOMAC-Score 1 year postoperatively. However, men had significantly better EQ-VAS results compared to women.

Joly D.A. et al. divided his study collective into three age categories: <55, 55–70 and >70 and obtained WOMAC and EQ-5D scores 3 and 12 months postoperatively after THA and TKA [18]. Patients over 70 years had significantly (*p* < 0.01) worse outcomes than patients between 55–70 years, as well as patients under 55 years. Our results with regard to age-correlated results of EQ-5D and WOMAC score 1 year postoperatively showed similar findings. Older patients have lower muscle quality and hip joint function after THA, which may be associated with locomotive dysfunction [41]. Based on our study findings, we state that clinical outcome decreases significantly with age. The incidence of TJA performed increases, especially in elderly patients; accordingly, geriatric complex treatment is becoming more important [42]. The combination of well-informed patients and implementation of geriatric co-management will reduce the rate of complications and the days in hospital [43,44].

## 5. Conclusions

We were able to demonstrate that preoperative anxiety/depression symptoms show significantly (*p* < 0.01) worse clinical outcomes (EQ-5D and WOMAC) 1-year-postoperatively after THA/TKA. Other possible outcome-influencing factors are higher age and ASA. ASA II and ASA III patients had significantly (*p* < 0.05) worse EQ-5D, EQ-VAS, and WOMAC scores than ASA I patients. Gender showed no significant (*p* > 0.05) difference regarding the EQ-5D or WOMAC-Score 1 year postoperatively. However, men had significantly better EQ-VAS results compared to women.

Our main goal regarding TJA-procedures should be a good clinical outcome. We should establish psychological and clinical assessments and therapies preoperatively, which might improve the degree of postoperative satisfaction in the future.

## Figures and Tables

**Table 1:** The relative and absolute frequencies of ASA score (*n* = 5156), gender (*n* = 5447) and age (*n* = 5447).

ASA		
	**Relative Frequency**	**Absolute Frequency**
1	13.91%	717
2	60.12%	3100
3	25.78%	1329
4	0.19%	10
5/6	0.00%	0
**Gender**		
Men	43.3%	2359
Women	56.59%	3088
**Age**		
<55 years	6.32%	344
55–70 years	54.93%	2992
>70 years	38.76%	2111

**Table 2:** The impact of preoperative anxiety/depression on EQ-5D values postoperatively: Median (IQR), minimum and maximum values.

EQ-5D Post-Operatively			
	Median (IQR)	Min	Max
not anxious/depressed	0.91 (0.83;1.00)	0.14	1
little anxious/depressed	0.91 (0.81;1.00)	0.11	1
moderately anxious/depressed	0.84 (0.73;1.00)	0.11	1
very anxious/depressed	0.83 (0.58;1.00)	0.14	1
extremely anxious/depressed	0.79 (0.49;1.00)	0.11	1

**Table 3:** EQ-5D Total, EQ-VAS, WOMAC Pain, WOMAC Stiffness, WOMAC Function and WOMAC Total overview of mean values, standard deviations and *p*-value. Divided by gender, age and ASA.

Post-OP	EQ-5D Total	EQ-VAS	WOMAC Pain	WOMAC Stiffness	WOMAC Function	WOMAC Total
Gender						
Male	0.86 (±0.17)	74.85 (±20.46)	3.37 (±3.48)	2.03 (±1.62)	14.50 (±12.43)	19.90 (±16.80)
Female	0.85 (±0.18)	72.88 (±22.39)	3.32 (±3.60)	2.00 (±1.69)	14.45 (±12.71)	19.77 (±17.23)
*p*-value	0.38	0.01	0.71	0.61	0.92	0.84
Age						
<55 Years	0.86 (±0.19)	77.20 (±20.54)	3.18 (±3.83)	2.06 (±1.83)	12.91 (±13.16)	18.15 (±18.12)
55–70 Years	0.87 (±0.16)	76.03 (±20.99)	3.15 (±3.40)	1.95 (±1.61)	13.56 (±11.99)	18.66 (±16.26)
>70 Years	0.83 (±0.19)	69.91 (±21.99)	3.69 (±3.62)	2.08 (±1.66)	16.32 (±13.01)	22.08 (±17.50)
*p*-value	<0.01	<0.01	<0.01	0.90	<0.01	<0.01
ASA (American Society of Anesthesiologists)						
ASA I	0.91 (±0.14)	81.41 (±19.13)	2.18 (±2.80)	1.72 (±1.55)	10.14 (±10.55)	14.04 (±14.17)
ASA II	0.86 (±0.16)	74.33 (±20.96)	3.33 (±3.51)	2.02 (±1.63)	14.31 (±12.31)	19.65 (±19.69)
ASA III	0.81 (±0.20)	68.33 (±22.88)	3.94 (±3.89)	2.13 (±1.78)	17.01 (±13.64)	23.09 (±18.53)
ASA IV	0.90 (±0.21)	75.67 (±23.55)	3.20 (±3.27)	2.20 (±2.17)	12.60 (±13.39)	18.00 (±18.15)
*p*-value	<0.01	<0.01	<0.01	<0.01	<0.01	<0.01

**Table 4:** The impact of preoperative anxiety/depression on Western Ontario and McMaster Universities Osteoarthritis Index (WOMAC) postoperatively: Median (IQR), minimum and maximum values.

WOMAC Postoperatively			
	Median (IQR)	Min	Max
not anxious/depressed	12.00 (5.00;26.00)	0	96
little anxious/depressed	15.00 (7.00;29.00)	0	96
moderately anxious/depressed	19.00 (9.00;34.00)	0	88
very anxious/depressed	18.00 (9.00;42.50)	0	85
extremely anxious/depressed	32.00 (19.00;51.50)	1	88

## Data Availability

Data is available at the institution of the authors.
